# Direct Oral Anticoagulants in Patients with Obesity and Atrial Fibrillation: Position Paper of Italian National Association of Hospital Cardiologists (ANMCO)

**DOI:** 10.3390/jcm10184185

**Published:** 2021-09-16

**Authors:** David Mocini, Stefania Angela Di Fusco, Edoardo Mocini, Lorenzo Maria Donini, Carlo Lavalle, Andrea Di Lenarda, Carmine Riccio, Pasquale Caldarola, Leonardo De Luca, Michele Massimo Gulizia, Fabrizio Oliva, Domenico Gabrielli, Furio Colivicchi

**Affiliations:** 1 U.O.C. Cardiologia Clinica e Riabilitativa, Presidio Ospedaliero San Filippo Neri, ASL Roma 1, 00135 Roma, Italy; stefaniaa.difusco@aslroma1.it (S.A.D.F.); furio.colivicchi@aslroma1.it (F.C.); 2 Department of Experimental Medicine, Sapienza University, 00161 Rome, Italy; edoardo.mocini@uniroma1.it (E.M.); lorenzomaria.donini@uniroma1.it (L.M.D.); 3 Department of Cardiovascular, Respiratory, Nephrological, Anesthesiological and Geriatric Sciences, “Sapienza” University of Rome, Policlinico Umberto I, 00161 Rome, Italy; carlo.lavalle@uniroma1.it; 4 S.C. Cardiovascolare e Medicina dello Sport, Azienda Sanitaria Universitaria Giuliano Isontina-ASUGI, 34128 Trieste, Italy; ccv@asugi.sanita.fvg.it; 5 UOSD “Follow up del paziente post acuto”, Dipartimento Cardiovascolare, Azienda Ospedaliera Sant’Anna e San Sebastiano, 81100 Caserta, Italy; carmine.riccio@tin.it; 6 U.O. Cardiologia-UTIC, Ospedale San Paolo, 70123 Bari, Italy; pascald@libero.it; 7 U.O.C. di Cardiologia, Dipartimento Cardio-Toraco-Vascolare, Azienda Ospedaliera San Camillo Forlanini, 00152 Roma, Italy; leo.deluca@libero.it (L.D.L.); dgabriellli@scamilloforlanini.rm.it (D.G.); 8 U.O.C. Cardiologia, Ospedale Garibaldi-Nesima, Azienda di Rilievo Nazionale e Alta Specializzazione “Garibaldi”, 95126 Catania, Italy; michele.gulizia60@gmail.com; 9 Fondazione per il Tuo cuore—Heart Care Foundation, 50121 Firenze, Italy; 10 1-Emodinamica, Unità di Cure Intensive Cardiologiche, Dipartimento Cardiotoracovascolare “A. De Gasperis”, ASST Grande Ospedale Metropolitano Niguarda, 20162 Milano, Italy; fabrizio.oliva@ospedaleniguarda.it

**Keywords:** obesity, atrial fibrillation, direct oral anticoagulants, DOACs, apixaban, dabigatran, edoxaban, rivaroxaban

## Abstract

The use of the direct oral anticoagulants dabigatran, rivaroxaban, apixaban and edoxaban (DOACs) offers some major advantages over warfarin and other vitamin K antagonists (VKAs). One advantage is the possibility to use a fixed dose in normal-weight patients, overweight patients and patients with obesity. However, the “one size fits all” strategy raised a concern regarding the possibility to undertreat patients with a high body mass index. No randomized controlled trials (RCTs) have ever compared VKAs and DOACs in this population. We analyzed data from the literature on DOAC pharmacokinetics and pharmacodynamics, results from the four pivotal phase III trials on non-valvular atrial fibrillation, retrospective observational studies and metanalyses. While we are aware of the limitation imposed by the absence of specific RCTs, we propose the position of the Italian Association of Hospital Cardiologists (ANMCO) on the use of DOACs in patients with obesity based on the existing evidence.

## 1. Introduction

Overweight and obesity are two conditions characterized by abnormal and excessive fat accumulation that may impair health [1]. Obesity is defined as a body mass index (BMI) of ≥30 kg/m^2^. Morbid, extreme or severe obesity is defined as a BMI of ≥40 kg/m^2^ [2].

The weight status classification associated with BMI categories is presented in Table 1.

BMI is the most used obesity measure in the scientific literature but has some limitations.

It cannot take into account body compositions, particularly excess fat mass, which is the main determinant of the clinical and functional consequences of obesity. Furthermore, BMI does not provide any information about fat location, which has a relevant impact on cardiovascular risk [3]. Despite its limitations, this measure is easy to apply, inexpensive and useful in predicting clinical outcome in epidemiological studies [4].

In Italy, 35% of the population is overweight, and more than 10% is obese [5].

In the United States of America, nearly 40% of adults met the criteria for obesity in 2017–2018, with approximately 9% meeting the criteria for grade 3 obesity [6].

In 2015, 107.7 million children and 603.7 million adults worldwide were estimated to have obesity [7]. The overall prevalence of obesity was 5% among children and 12% among adults [6].

Obesity and atrial fibrillation (AF) are closely related through epidemiological and pathophysiological relationships. Atrial fibrillation (AF) is the most common sustained arrhythmia in adult individuals [8], accounting for a substantial increase in stroke risk and reduction in quality of life [9,10]. Its prevalence is closely age-dependent, increasing from 1.3% in males (M) and 1.7% in females (F) aged 55–59 years to 24.2% (M) and 16.1% (F) in people aged >85 years [11]. In a recent publication, the World Heart Federation summarized a prevalence of 1–3% in the general population, up to 9% in people aged ≥65 years and up to 17% in people ≥80 years old [12].

The increasing age of the population and the increasing prevalence of other AF risk factors such as hypertension, coronary artery disease, diabetes mellitus, heart failure, chronic kidney disease, obesity, chronic obstructive pulmonary disease and obstructive sleep apnea explain the higher burden of AF that is expected in the coming decades [11,12,13,14].

AF and obesity patient populations partially overlap, and both are experiencing epidemic growth [8,11,12,14]. Subjects with obesity have a 51% increased risk of AF occurrence [9,15] and a higher rate of AF recurrence after catheter ablation [16]. The increase in atrial size, the reduction in ventricle diastolic function, autonomic dysfunction, enhanced neurohormonal activation, greater epicardial fat thickness and the chronic subclinical inflammation can partially explain the AF prevalence in patients with obesity with respect to the general population [15,17].

Since 2009, the introduction of four new direct oral anticoagulants (DOACs) into clinical practice has changed anticoagulant therapy for non-valvular AF.

Dabigatran, rivaroxaban, apixaban and edoxaban offer some improvements in both efficacy and safety [18,19,20,21,22,23,24]. Furthermore, the faster onset of action, reduced drug-drug and drug–food interactions, reduced need for frequent blood tests and more predictable activity are leading to widespread utilization.

For these reasons, DOACs are generally considered a better option in non-valvular AF, and current guidelines recommend them over warfarin or other vitamin K antagonists (VKAs) [18,19,20], despite the fact that randomized controlled trials (RCTs) have shown only non-inferiority against warfarin [21,22,23,24].

However, some controversy still exists. No RCTs have been specifically conducted in patients with obesity. Available data are derived from post hoc analyses, metanalyses, retrospective analyses, observational studies and small non-randomized studies.

None of these four drugs need dose adjustment in patients with overweight and obesity, while a reduced dose must be considered in low-body weight patients on apixaban or edoxaban.

Pharmacokinetic (PK) data in extreme obesity support the suspicion that patients with a high BMI could be underexposed to DOACs and thus undertreated [25].

Obesity could affect drug pharmacokinetics by increasing the volume of distribution and altering drug clearance as well as other pharmacodynamic effects. Traditional anticoagulants are dosed either based on laboratory testing (VKAs) or by weight (as is the case for heparin). Thus, when fixed-dose DOACs became the mainstay of anticoagulation, many clinicians were concerned about their efficacy and safety in patients who have obesity, since dosing is not weight-based, and laboratory monitoring is not commonly performed.

The aim of this position paper is to review the available literature and propose the position of the Italian Association of Hospital Cardiologists (ANMCO) regarding the use of DOACs in patients with obesity and AF. In this paper, non-valvular atrial fibrillation refers to AF in the absence of a mechanical prosthetic heart valve or moderate to severe mitral stenosis.

## 2. Pharmacokinetics and Obesity

The pharmacokinetics (PK) and pharmacodynamics (PD) of DOACs have already been reported in detail in the scientific literature [26,27,28,29]. Table 2 shows some PK properties of DOACs.

PK differences in patients with obesity, as compared to those with normal weight, can potentially induce a different drug exposure and therefore a different magnitude of the anticoagulation effect [25].

Drug absorption is a complex event that depends on drug features and specific properties of the host system. Gut permeability [34], liver metabolism [35] and gastric emptying velocity [36] can be different in patients with morbid obesity.

Drug distribution is the most controversial PK topic. Lipophilic drugs are expected to easily diffuse in fat tissue, and thus a higher volume of distribution (Vd) should be found in patients with extreme obesity. This is true for diazepam [37] but not for propofol [38], both lipophilic drugs. Conversely, when using a hydrophilic drug, an increase in total body weight (TBW) is not followed by an increase in Vd, resulting in a reduction in the Vd/TBW ratio. This is true for ranitidine [39] but not for vancomycin [40], both hydrophilic drugs. For these reasons, predicting the drug distribution with a simple evaluation of chemical drug features is challenging.

Drug metabolism and excretion are mainly controlled by hepatic and renal functions. These two variables together with Vd determine the drug elimination half time.

Vd [37,38], renal elimination [38] and hepatic metabolism [25] can differ in patients with a high BMI with respect to those with a lower BMI, and between the four DOACs.

In severe obesity, the glomerular filtration rate (GFR) and renal plasma flow (RPF) were 51% and 31% greater than these values in control subjects [41].

In patients with obesity, an increase in cytochrome P450 2E1 (CYP2E1) activity and phase II conjugation activity has been observed [25].

For these reasons, several drug regimens are weight-adjusted, though this is not the case with DOACs, which are approved in fixed doses in patients with a high BMI [30,31,32,33].

Kubitza et al. [42], in a single-center, randomized, single-blind, placebo-controlled, parallel-group study, detected no differences in the rivaroxaban maximum plasma drug concentration (Cmax), area under the plasma concentration–time curve (AUC) or activated factor X (FXa) levels between healthy subjects weighting >120 kg and those with a weight of 70–80 kg. Due to the small influence of body weight on rivaroxaban plasma concentrations (less than 25%), the package insert reports that no dose adjustment is necessary [39].

Barsam et al. [43] evaluated the impact of BMI on rivaroxaban exposure in a real-world population. They concluded that weight had no relevant effect on rivaroxaban exposure.

Using data from seven clinical trials including 4918 patients with different approved indications of rivaroxaban, Willmann et al. [44] demonstrated that renal function was the main driver of rivaroxaban exposure, whereas the efficacy and safety profile were maintained across subgroups classified according to age, weight and BMI [44].

Liesenfeld et al. [45] analyzed data from the Randomized Evaluation of Long-Term Anticoagulation Therapy (RE-LY) study and found a similar result for dabigatran. The AUC was not affected by weight. Every 1 kg increase over a BMI median value of 80 kg was associated with an increase of 0.77% in Vd. This change had only a minor influence on the concentration–time profile and the overall exposure [45].

In a phase I study, Upreti et al. [46] investigated apixaban PK in patients with a low body weight (<50 kg), as compared with a reference body weight (65–85 kg), and high body weight (>120 kg). They described a 27% and 20% increase in mean apixaban *C*max and AUC extrapolated to infinity, respectively, in low-weight patients when compared with the reference body weight patients. Conversely, the same parameters were reduced by 31% and 23% in higher-BMI subjects with a significant inverse relationship between apixaban exposure and both body weight and BMI. Anti-factor Xa activity showed a direct linear relationship with the apixaban plasma concentration. In light of the small slope, the authors considered the relationship between BMI and drug exposure “unlikely to be clinically meaningfully”.

In a retrospective study on 38 patients with obesity (median body weight 132.5 kg), Piran et al. [47] analyzed dabigatran, rivaroxaban and apixaban peak plasma concentrations. All patients but two (95%) had peak plasma concentrations higher than the expected median trough level. The two patients were taking dabigatran.

Boriani et al. [48] analyzed data from the Effective Anticoagulation with Factor Xa Next Generation in Atrial Fibrillation–Thrombolysis in Myocardial Infarction 48 (ENGAGE AF TIMI 48) study and found that trough edoxaban plasma concentrations measured at steady state did not differ across BMI categories. The drug concentration and FXa inhibition were similar across the extremes of body weight.

From a theoretical point of view, DOACs with a low Vd, high protein binding and limited renal elimination should be less affected by body weight [49]; however, many other poorly understood elements can modify the final drug exposure such as comorbidities involving organs implicated in drug elimination, such as the liver and kidney.

Dosing the plasma drug concentration at steady state could be a simple solution to solve the problem, but most guidelines [18,19,20,50] have pointed out that it is not always feasible for various reasons, including the lack of clear correlation between drug levels, effective anticoagulation and outcome.

For example, in the Apixaban for Reduction in Stroke and Other Thromboembolic Events in Atrial Fibrillation (ARISTOTLE) trial, patients receiving a lower apixaban dose compared with those receiving a full dose had a lower median AUC at steady state (2720 ng/mL vs. 3599 ng/mL; *p* < 0.0001) but the same coagulations biomarkers, such as D-dimer and prothrombin fragment 1 + 2 levels, and clinical outcome [51].

Another limitation of dosing plasma drugs has been emphasized by Chan et al. [52], who analyzed dabigatran levels in 100 patients. They found high inter-patient variability (geometric coefficient of variation (gCV), 51–64%) and intra-patient variability (gCV, 32–40%). Nevertheless, similar medians and distributions of drugs levels were observed both in the 110 mg and 150 mg groups. The authors concluded that Hemoclot^®^ measurement was not superior to clinical judgment in identifying patients with high or low drug exposure.

In conclusion, the translation of data from PK studies to the clinical setting is difficult and not always possible. Generally, weight is not considered a relevant factor for drug exposure and its anticoagulant activity.

Physicians should be aware of patients with a creatinine clearance superior to 95 mL/min, a frequent occurrence in patients with a high BMI. Edoxaban should be avoided due to reduced efficacy in non-valvular atrial fibrillation in patients with this condition, as reported in the USA package insert [53].

## 3. Randomized Prospective and Controlled Phase III Studies

Up to now, to our best knowledge, no RCTs have been conducted comparing DOACs and warfarin specifically in patients with obesity and atrial fibrillation. Available data come from post hoc analysis of phase III trials.

Data from these studies have been reported in the International Society of Thrombosis and Haemostasis (ISTH) guidelines [50] (except for edoxaban), but the quality of the information obtained, as calculated by the Jadad score, is low due to the differences in the weight cut-off and BMI stratification used in the studies included.

Two ARISTOTLE study post hoc analyses have been conducted using different types of patient stratification [54,55]. Both reached a similar conclusion: apixaban is always at least non-inferior compared with warfarin irrespective of weight class.

Balla et al. [56], in a post hoc analysis of the Rivaroxaban Once Daily Oral Direct Factor Xa Inhibition Compared with Vitamin K Antagonism for Prevention of Stroke and Embolism Trial in Atrial Fibrillation (ROCKET AF) trial, found a reduced risk of stroke for obese patients with a BMI of ≥35 compared to that of normal-weight patients in both the rivaroxaban and warfarin groups, supporting the obese paradox hypothesis. No difference was detected in different weight classes according to drug treatment.

Boriani et al. [48] investigated efficacy and safety in patients enrolled in the ENGAGE AF-TIMI 48 trial without finding any difference across BMI groups. Edoxaban had similar PK (trough peak at steady state and anti-factor Xa activity) across the BMI range investigated. Obesity was present in more than 40% of the ENGAGE AF-TIMI 48 population.

Ezekowitz et al. [57] presented an analysis of data from The Randomized Evaluation of Long-Term Anticoagulation Therapy (RE_LY) by subdividing patients into three groups: low BMI (≤22.5 kg/m^2^, 1865 patients), mid-range BMI (22.5 to 36 kg/m^2^, 14,435 patients) and high BMI (>36 kg/m^2^, 1787 patients). No differences in efficacy or safety were found in the high-BMI group between dabigatran and warfarin.

In conclusion, about 24,000 patients with obesity were included in the four phase III registration trials (Table 3). The use of different cut-offs for obesity among the four trials produced low-quality data. With these limitations, data from post hoc analyses of phase III studies do not show a difference in efficacy or safety in patients with morbid obesity with respect to normal-weight patients.

## 4. Metanalyses

Metanalyses have investigated the effects of different weight classes compared to normal-weight patients on thromboembolic and bleeding events [58,59,60]. Evaluating RCT data, Proietti et al. [58] found an inverse association between BMI classes and outcomes, with a lower risk of stroke/systemic embolism and major bleeding in both overweight and obese patients compared to normal-weight patients. Similar findings were obtained with subsequent metanalyses [59,60] that included both RCTs and observational data.

These observations are the basis of the so-called “obesity paradox” hypothesis: counterintuitive observations suggesting that patients with overweight or obesity may have a better prognosis than healthy-weight patients in terms of cardiovascular events [61,62,63,64].

In metanalyses including data from RCTs only, DOAC treatment resulted in efficacy and safety at least comparable to warfarin in overweight and obese patients [58,59,65,66].

Both Wang et al. [66] and Zhou et al. [59] analyzed efficacy and safety in different BMI classes (by using different cut-offs), confirming a significantly better efficacy profile of DOACs compared to warfarin, without differences in major bleeding.

Additional metanalyses evaluated data from both RCTs and real-life studies [60,67,68]. Grymonprez et al. [68] analyzed stroke/systemic embolism, major bleeding, all-cause mortality, intracranial bleeding and gastrointestinal bleeding in DOACs vs. VKAs in class 1–2 (BMI ≥ 30 kg/m^2^) and class 3 (BMI ≥ 40 kg/m^2^) obese patients.

The analysis highlighted a better efficacy and safety of DOACs over VKAs in terms of stroke, systemic embolism and major bleeding in both groups and an even greater benefit in terms of the intracranial hemorrhage (ICH) incidence rate [68]. Notably, the significant reduction in stroke, systemic embolism and major bleeding events in class 3 obese patients was principally driven by data from observational studies. Another metanalysis [58] on patients with a BMI of ≥40 kg/m^2^ found no significant reduction in stroke or systemic embolism, but a significant reduction in major bleeding. Additionally, in this analysis, the results were driven by real-life studies.

Following an initial metanalysis including two post hoc analyses of the ARISTOTLE trial and three retrospective studies [67] with similar results to the previously mentioned analyses, Kido et al. [69] published an additional study using the same source data that compared the effect of rivaroxaban vs. apixaban. The results demonstrated no significant differences between the two molecules.

## 5. Recent Large Studies on DOACs in Patients with Obesity

Recently, Barakat et al. [70] presented a retrospective evaluation of 36,094 consecutive patients receiving DOACs or warfarin for non-valvular atrial fibrillation (NVAF) in a single center between 1 January 2010 and 31 May 2018. Patients were categorized according to their BMI into four groups: BMI < 18.5 kg/m^2^, BMI 18.5 to <30 kg/m^2^, BMI 30 to <40 kg/m^2^ and BMI ≥ 40 kg/m^2^. Furthermore, patients were divided according to the type of anticoagulant prescribed: dabigatran, rivaroxaban, apixaban, edoxaban or warfarin.

Considering all patients receiving DOACs as a group, the outcome of 17,640 patients in the treatment with warfarin was compared with that of 18,454 patients receiving DOACs: 1754 patients on warfarin were morbidly obese vs. 2170 on DOACs.

BMI was not an independent predictor of ischemic stroke, significant bleeding or hemorrhagic stroke. Similar results were obtained considering BMI as a categorical or a continuous variable. Increasing BMI was associated with a small but statistically significant reduction in the risk of all-cause mortality.

As compared with warfarin, DOACs showed improved safety and effectiveness across all BMI groups, including underweight and morbidly obese patients, at 3.8 years of follow-up. However, this observational study may have some limitations related to uncontrolled confounders.

In the Anticoagulants for Reduction In Stroke: Observational Pooled analysis on Health outcomes And Experience of Patients (ARISTOPHANES) study, Deitelzweig et al. [71] analyzed data from Centers for Medicare and Medicaid Services and four US commercial claims databases. After applying selection criteria, they identified 88,461 patients with obesity, of whom 39.5% had morbid obesity. They used propensity score matching to create six groups comparing each DOAC against warfarin and each DOAC against other DOACs (apixaban vs. dabigatran, apixaban vs. rivaroxaban and dabigatran vs. rivaroxaban). Edoxaban was excluded due to a small sample size. In summary, DOAC use was associated with an outcome at least not inferior to and, in some cases, better than warfarin.

Berger et al. [72] assessed the effectiveness and safety of rivaroxaban as compared to warfarin in a retrospective observational weighted-cohort study. Inverse probability of treatment weighting, based on the propensity score, was used to create two populations from the insurance database. The first one was composed of 10,555 patients receiving rivaroxaban, and the second one was composed of 5080 receiving warfarin. Rivaroxaban was associated with a 26% lower risk of stroke/systemic embolism compared with warfarin and the same risk of bleeding over the 36-month follow-up period.

## 6. Guidelines

We identified seven scientific society guidelines or position papers on AF management [18,19,20,50,73,74,75].

The most exhaustive guidelines on DOAC use in overweight, obese and extremely obese patients are from the Scientific and Standardization Committee (SSC) of ISTH [50] and the 2018 [73] and 2021 [19] European Heart Rhythm Association (EHRA) Practical Guide on the Use of Non-Vitamin K Antagonist Oral Anticoagulants in Patients with Atrial Fibrillation.

The guidelines of the SSC of the ISTH published in 2016 were the first society statement to raise concern about the use of DOACs in patients with obesity [50]. Guidance statements can be summarized in three items.

1. *The recommendation* to use standard dosing in people with a weight equal to or less than 120 kg or a BMI equal to or less than 40 kg/m^2^;
2. *The suggestion* to not use DOACs in patients with a weight of more than 120 kg or a BMI of more than 40 kg/m^2^;
3. *The suggestion* to evaluate, in patients with the characteristics of point 2, a drug-specific peak and trough level using a specific assay depending on the drugs used.

In 2018, the EHRA Practical Guide on the use of non-vitamin K antagonist oral anticoagulants in patients with atrial fibrillation [73] reported similar guidance. In extreme obesity, the use of VKAs should be considered, and the evaluation of DOAC drug peak and trough levels needs special caution and expertise.

In 2018, the ESC working group on thrombosis assembled a task group that published a consensus statement on antithrombotic management related to body mass [75]. The conclusions were similar to the 2016 ISTH recommendations, with an additional warning, pointing out that data are insufficient for apixaban, dabigatran and edoxaban in class 2 obesity.

In the 2019 AHA/ACC/HRS Focused Update of the 2014 AHA/ACC/HRS Guideline for the Management of Patients With Atrial Fibrillation [18], specific management of obese patients was not recommended or suggested, though it was recommended to measure DOAC serum levels in patients with a BMI of >35 kg/m^2^ or a weight of >120 kg.

**Figure 1:**
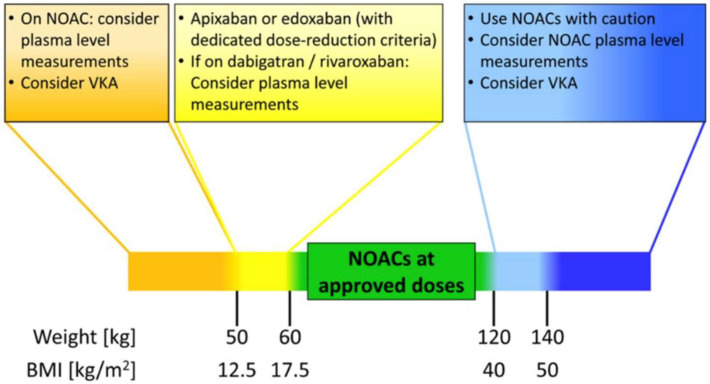
Legend. BMI: body mass index; NOAC: non-vitamin K antagonist oral anticoagulant; VKA: vitamin K antagonist. From Europace with permission [19].

The conclusions of the most recent EHRA practical guide on non-VKA oral anticoagulants are summarized in Figure 1 [19].

Briefly, at a BMI of 17.5–40 kg/m^2^ and a weight of 50–120 kg, DOACs can be safely used as usual. Between a BMI of 40 and 50 kg/m^2^ and a weight of 120 and 140 kg, the use of DOACs can be considered, but data are less robust. Over these upper limits, the need for careful individual clinical evaluation and DOAC plasma level measurements increases. According to international society recommendations, the use of VKAs may be considered a better option in this setting, and treatment individualization is mandatory.

In conclusion, guidelines direct special attention to DOAC use in people with extreme obesity. DOACs can be used, but individualized management should guide clinicians, possibly with the help of DOAC plasma level measurement. Over a BMI of 50 kg/m^2^ and a weight of 140 kg, scarce data are available. DOAC plasma level measurements or the use of VKAs may be reasonable [19].

Plasma level measurements may aid clinicians in decision making but should only be used under the guidance of an expert team.

## 7. Discussion

The use of DOACs offers some major advantages with respect to warfarin and other VKAs [18,19,20].

One advantage is the possibility to use a fixed dose in patients with a high BMI, irrespective of patient size.

However, the “one size fits all” strategy has raised a reasonable concern since different PKs in extreme BMI could induce a different drug exposure and thus a different anticoagulation effect [50].

To test this hypothesis, an RCT in patients with extreme obesity should be conducted, comparing each DOAC against VKAs. Assuming a power of 80% and an alpha error of 0.05, a non-inferiority study would need at least 3000 patients in the DOAC arm and 3000 patients in the warfarin arm. It is unlikely that such a study can be carried out soon.

However, PK data from populations with obesity, when available, are reassuring about drug exposure [38,39,40,41,42,43,44,45,46,47,48]. Moreover, the outcome is always at least equal, if not better, in large retrospective and observational studies [57,58,59,60,61,62,63,64,65,66,67,68,69,70,71,72]. BMI is not a relevant covariate when tested [43,44,47,48,70].

A different number of studies have been performed for each DOAC. Rivaroxaban and apixaban are the most studied [70].

Additionally, the use of warfarin in populations with obesity presents some problems and limitations. VKAs suffer from important food–drug interactions [76].

There is an up to 40% prevalence of binge eating disorder or food addiction in extreme obesity [77,78], which could lead to an unpredictable increase in vitamin K intake, resulting in a reduced anticoagulation activity. Drug–drug interactions are also relevant [76].

Many patients with extreme obesity are affected by some deficits in executive function, such as inhibitory control or attentional bias [79]. The simplification of drug treatment can significantly increase patient engagement and adherence.

The lack of prospective, randomized and controlled studies comparing each DOAC with VKAs in populations with extreme obesity means there is limited knowledge on this subject. However, knowledge is also limited in other important settings. For example, the use of DOACs in the specific setting of cardioversion has been evaluated in three deliberately designed undersized RCTs [80,81,82].

These three studies were undersized because from 20,000 to 48,000 patients would have been necessary to test non-inferiority, making it impossible. For this reason, other criteria different from powered RCTs have been considered acceptable.

Furthermore, in patients affected by acute coronary syndrome, clopidogrel, ticagrelor, prasugrel and aspirin are commonly used in extremely obese patients, though no RCTs are available comparing each drug to the previous standard of care [75].

## 8. Conclusions and ANMCO Position

It is reasonable to consider the use of DOACs as safe and effective in all weight classes.

Considering our knowledge limitations, DOACs should be preferred unless specific reasons to the contrary are identified. Rivaroxaban and apixaban have more data and stronger evidence. Edoxaban should be avoided if a patient has a creatinine clearance superior to 95 mL/min, a frequent possibility with extreme BMI, as specified in the US package insert [53].

Treatment in patients with a BMI equal or superior to 50 kg/m^2^ must be individualized and determined together with a multidisciplinary team since data are lacking and special expertise is needed.

Checking drug-specific peak and trough levels is currently not a completely satisfactory solution for both theoretical and practical reasons, such as the lack of a clear correlation between drug levels, effective anticoagulation and outcome. Measurement of DOAC serum levels can be considered in specific situations if expertise is available, but current knowledge does not suggest routine daily use.

## Figures and Tables

**Table 1:** Weight categories according to adult body mass index.

Categories	BMI (Kg/m^2^)
Underweight	<18.5
Healthy weight	18.5 to <25
Overweight	25 to <30
Obesity	30 or higher
Class 1 obesity	30 to <35
Class 2 obesity	35 to <40
Class 3 obesity	40 or higher

Legend. Weight status classification associated with body mass index (BMI) categories. Class 3 obesity is also called severe, extreme, or morbid obesity.

**Table 2:** Pharmacokinetics properties of DOACs.

	Dabigatran [26,30]	Rivaroxaban * [27,31]	Apixaban [28,32]	Edoxaban # [29,33]
Vd (L)	60–70	50	21	107
Pbp (%)	34–35	92–95 Al	87 Al	55
UE (%)	80	66	24.5	50
Food–drug interaction	no	yes	no	no
CYP isoenzyme interactions	no	yes	yes	yes
F (%)	6,5	80–100	50	63
Prodrug	yes	no	no	no
Non-renal clearance (%)	20	35	73	50
Time to peak (h)	3	2–4	3	2–4
Half life elimination	15	5–13	12	10–14
Plasma level expected (19) (ng/mL)				
Peak	52–382	178–343	69–321	101–288
Trough	28–215	12–137	34–230	12–43
Dialysability (no)	partially	no	no	no

Legend. DOAC clinical pharmacokinetic features. Vd: volume of distribution, L: liters; Pbp: plasma binding protein; UE: urine excretion; F: bioavailability; Al: albumin, * 20 mg, # 60 mg.

**Table 3:** Percentage of patients with obesity included in the four main phase III trials on DOACs and atrial fibrillation.

Reference	Data Source	Obesity Cut-Off	Obese Subjects (*n*)	Total Population (*n*)	Obese %
Connolly, S.J. et al. [21]	RE-LY	≥100 kg	3099	18,113	17.1
Ezekowitz, M.E. et al. [57]	RE-LY	>36 kg/m^2^	1787	18,113	9.9
Patel, M.R. et al. [22]	ROCKET AF	>90 kg	3977	14,171	28.1
Balla, S.R. et al. [56]	ROCKET AF	≥30 kg/m^2^	5206	14,030	37.1
Patel, M.R. et al. [22]	ROCKET AF	>35 kg/m^2^	1871	14,171	13.2
Hohnloser, S.H. et al. [54]	ARISTOTLE	>120 kg	982	18,139	5.4
Sandhu, R.K. et al. [55]	ARISTOTLE	≥30 kg/m^2^	7159	17,913	40
Boriani G. et al. [48]	ENGAGE AF TIMI 48	≥30 kg/m^2^	8457	21,028	40.3

Legend. DOACs: direct oral anticoagulants. RE-LY indicates Randomized Evaluation of Long-Term Anticoagulation Therapy; ROCKET AF, Rivaroxaban Once Daily Oral Direct Factor Xa Inhibition Compared with Vitamin K Antagonism for Prevention of Stroke and Embolism Trial in Atrial Fibrillation; ARISTOTLE, Apixaban for Reduction in Stroke and Other Thromboembolic Events in Atrial Fibrillation; ENGAGE AF TIMI 48, Effective Anticoagulation with Factor Xa Next Generation in Atrial Fibrillation–Thrombolysis in Myocardial Infarction 48.

## Data Availability

Not applicable.
